# Comparative Genomics and Description of Putative Virulence Factors of *Melissococcus plutonius*, the Causative Agent of European Foulbrood Disease in Honey Bees

**DOI:** 10.3390/genes9080419

**Published:** 2018-08-20

**Authors:** Marvin Djukic, Silvio Erler, Andreas Leimbach, Daniela Grossar, Jean-Daniel Charrière, Laurent Gauthier, Denise Hartken, Sascha Dietrich, Heiko Nacke, Rolf Daniel, Anja Poehlein

**Affiliations:** 1 Department of Genomic and Applied Microbiology & Göttingen Genomics Laboratory, Institute of Microbiology and Genetics, Georg-August-University of Göttingen, 37077 Göttingen, Germany; mdjukic@posteo.de (M.D.); aleimba@gmx.de (A.L.); denise.hartken@uni-goettingen.de (D.H.); sdietri@gwdg.de (S.D.); hnacke@gwdg.de (H.N.); rdaniel@gwdg.de (R.D.); 2 Molecular Ecology, Institute of Biology, Martin-Luther-University Halle-Wittenberg, Hoher Weg 4, 06099 Halle (Saale), Germany; erler.silvio@gmail.com; 3 Swiss Bee Research Center, Agroscope, 3003 Bern, Switzerland; daniela.grossar@gmail.com (D.G.); jean-daniel.charriere@agroscope.admin.ch (J.-D.C.); ruchersastet@club.fr (L.G.); 4 Department of Ecology and Evolution, Biophore, UNIL-Sorge, University of Lausanne, 1015 Lausanne, Switzerland

**Keywords:** European foulbrood, comparative genomics, pathogenesis, *Melissococcus plutonius*, toxin, virulence factor, *Apis mellifera*, brood disease, host-parasite interaction

## Abstract

In Europe, approximately 84% of cultivated crop species depend on insect pollinators, mainly bees. *Apis mellifera* (the Western honey bee) is the most important commercial pollinator worldwide. The Gram-positive bacterium *Melissococcus plutonius* is the causative agent of European foulbrood (EFB), a global honey bee brood disease. In order to detect putative virulence factors, we sequenced and analyzed the genomes of 14 *M. plutonius* strains, including two reference isolates. The isolates do not show a high diversity in genome size or number of predicted protein-encoding genes, ranging from 2.021 to 2.101 Mbp and 1589 to 1686, respectively. Comparative genomics detected genes that might play a role in EFB pathogenesis and ultimately in the death of the honey bee larvae. These include bacteriocins, bacteria cell surface- and host cell adhesion-associated proteins, an enterococcal polysaccharide antigen, an epsilon toxin, proteolytic enzymes, and capsule-associated proteins. In vivo expression of three putative virulence factors (endo-alpha-*N*-acetylgalactosaminidase, enhancin and epsilon toxin) was verified using naturally infected larvae. With our strain collection, we show for the first time that genomic differences exist between non-virulent and virulent typical strains, as well as a highly virulent atypical strain, that may contribute to the virulence of *M. plutonius*. Finally, we also detected a high number of conserved pseudogenes (75 to 156) per genome, which indicates genomic reduction during evolutionary host adaptation.

## 1. Introduction

The Western honey bee (*Apis mellifera*) is the most important commercial pollinator worldwide [1,2,3]. Approximately 84% of crop species cultivated in Europe depend on insect pollinators, mainly bees [4]. Furthermore, honey bees contribute to the pollination of wild plants and are known for their production of economical and medical relevant products such as honey, beeswax, pollen, beebread, propolis, royal jelly, and apitoxin. European foulbrood (EFB) is one of the major bacterial diseases of honey bees [5]. The etiological agent of EFB is *Melissococcus plutonius* [6], which is a Gram-positive, microaerophilic and lanceolate coccus. In general, EFB mainly affects the unsealed brood, and infected honey bee larvae usually die when they are four to five days old [5]. Infection occurs by ingestion of larval food contaminated with *M. plutonius*. Subsequently, *M. plutonius* colonizes the larval gut. Dying larvae are displaced to the wall of the comb cell, often turn yellow and finally take on a brownish color, as they decompose after death [7]. In contrast to *M. plutonius* typical strains, atypical strains (detected mainly in the UK and Japan, [8]) display a higher virulence and are not fastidious, meaning that they are able to grow aerobically on some potassium salt-supplemented media. They do not require potassium phosphate for growth, show β-glucosidase activity and hydrolyze esculin [9]. Furthermore, in contrast to atypical strains, typical strains often lose their virulence after a few cultivation steps in vitro [6,9], which hinders the investigation of virulence factors under laboratory conditions. Nevertheless, variance in virulence related to different *M. plutonius* phylogenetic groups, more precisely, sequence types that can be grouped to clonal complexes (CC), has recently been shown [10]. The authors showed that atypical strain (CC12) was the most virulent followed by the typical strain of CC3, and a typical strain ofCC13 was non-virulent [10]. Field pathology data confirmed mostly the decreasing virulence from CC12 and CC3 to CC13 [8]. Until now, virulence factors for *M. plutonius* were not described, and therefore the pathogenicity mechanism remains unclear.

In this study, we investigate if phylogeny-related virulence is a consequence of variance in the gene content. For this purpose, we present the whole genome sequences of 12 *M. plutonius* strains and a comparative genome analysis with type strain ATCC 35311 [11] and atypical strain DAT561 from Japan [12]. We identified putative virulence factors of *M. plutonius* that might play an important role in EFB pathogenesis. Additionally, we investigated the expression profiles of three putative virulence factors in naturally EFB-infected larvae. In this way, the study contributes to the prospective development of a conceptual pathogenicity model and the underlying molecular mechanisms of EFB disease.

## 2. Materials and Methods

### 2.1. Origin of Melissococcus plutonius Strains

*M. plutonius* ATCC 35311 (type strain) was originally isolated in United Kingdom and represents a typical strain according to Bailey and Collins [13]. Strain DAT561 represents an atypical strain and originated from Japan [12]. The typical strains 764-5B and 765-6B were isolated from EFB outbreaks in Norway and all other typical strains (B5, H6, L9, S1, 21.1, 49.3, 60, 82, 90.0, and 119) from Swiss EFB outbreaks. Isolates B5, H6, and L9 originate from the same Swiss EFB outbreak and the same EFB-infected larva. Strains 21.1, 49.3, 60, 82, 90.0, and 119 were isolated from different EFB outbreaks. *M. plutonius* S1 is a derivative of strain 49.3 and was isolated after five cultivation steps.

### 2.2. Growth Conditions and Isolation of DNA from Melissococcus plutonius

Honey bee larvae with clinical EFB symptoms were collected from the aforementioned EFB outbreaks in Switzerland and Norway, and dissected under sterile conditions. Isolates were prepared from diseased larvae mixed with Bailey medium [14]. Larval smears were streaked on solidified Bailey agar. Single colonies were selected after anaerobic incubation at 35 °C for five days. All colonies were verified to be *M. plutonius* using species-specific 16S rRNA primers (Table S1). Genomic DNA of *M. plutonius* was extracted from cells in the exponential growth phase using the Epicentre MasterPure DNA purification kit (Epicentre, Madison, WI, USA) as recommended by the manufacturer.

### 2.3. Genome Sequencing, Assembly and Annotation

Whole-genome shotgun sequencing of *M. plutonius* strain S1 was performed with a combined sequencing approach using a Genome Analyzer IIx (Illumina, San Diego, CA, USA) and a 454 GS-FLX Titanium XL+ system (GS70 chemistry, Roche Life Science, Mannheim, Germany). 454-shotgun and Illumina Nextera XT shotgun libraries were prepared as recommended by the manufacturers. Sequencing resulted in 149,969 total 454 shotgun reads and 869,292 Illumina 112 bp paired-end reads, which were used for a hybrid assembly with MIRA v3.4.0.1 [15]. An average coverage of 67-fold was achieved (Table S2). Editing of the resulting contigs was performed with GAP4, as part of the Staden software package [16]. Misassembled regions caused by repetitive sequences were resolved, and closure of remaining gaps was performed by PCR reactions and subsequent Sanger sequencing. Whole-genome shotgun sequencing of *M. plutonius* isolates B5, H6, L9, 21.1, 60, 82, 764-5B, and 765-6B was carried out by generating paired-end libraries (2 × 112 bp) with the Nextera XT library preparation kit and employing the Genome Analyzer IIx as recommended by the manufacturer (Illumina, San Diego, CA, USA). Strains 49.3, 90.0 and 119 were sequenced at the Beijing Genomics Institute (Shenzhen, Guangdong, China) with an Illumina Hiseq2000 (Illumina, San Diego, CA, USA) by employing 90 bp paired-end reads. After read trimming and quality check using Trim_Galore v0.3.7 (http://www.bioinformatics.babraham.ac.uk/projects/trim_galore/) and FastQC v0.11.2 (https://www.bioinformatics.babraham.ac.uk/projects/fastqc/), respectively, a de novo assembly of the paired-end Illumina reads was done with the SPAdes software 3.7.0 [17]. Bacteria genome sizes were estimated on the outcome of the genome assemblies (Table S2). All small fragments (>0.5 kb) have been submitted along with larger scaffolds and plasmids. Genomic fragments of 0.5 to 3 kb usually consist of RNA clusters, transposases and repetitive elements. Therefore, we expect no larger gaps or missing sequence information in the reported genomes. 

Prokka v1.9 [18] was used for automatic gene prediction and annotation. Annotation was manually curated by employing BLASTP and the Swiss-Prot [19], TrEMBL [20] and InterProScan 5 databases [21], and the IMG-ER (integrated microbial genomes-expert review) system [22]. The rRNA and t-RNA genes were identified with RNAmmer v1.2 [23] and tRNAscan-SE v1.3.1 [24], respectively.

The genome sequences of *M. plutonius* ATCC 35311 (BioProject: PRJDA61383) and DAT561 (BioProject: PRJDA73165) were obtained from NCBI and re-annotated as described above for comparative genome analyses (Data S1).

Genome quality was tested by means of estimating genome completeness, contamination and heterogeneity by using CheckM [25]. The 14 *M. plutonius* genomes were compared to 55 genomes of the family *Enterococcaceae*, the next relatives of *M. plutonius*. CheckM compares absence, presence and total copy number for 542 markers with 226 collocated gene sets that are ubiquitous and single-copy within the *Enterococcaceae* phylogenetic lineage.

DNA sequences obtained and GenBank submissions: The genome sequences reported in this paper have been deposited in the GenBank database under accession numbers: JSAY00000000 (*M. plutonius 21.1*), JSBA00000000 (*M. plutonius* 49.3), JSBE00000000 (*M. plutonius* 60), JSBF00000000 (*M. plutonius* 82), JSAZ00000000 (*M. plutonius* 90.0), JSBB00000000 (*M. plutonius* 119), JSAW00000000 (*M. plutonius* B5), JSBC00000000 (*M. plutonius* H6), JSBD00000000 (*M. plutonius* L9), CP006683-CP006684 (*M. plutonius* S1), JSAV00000000 (*M. plutonius* 764-5B), JSAX00000000 (*M. plutonius* 765-6B).

### 2.4. Genome Analyses

The sequence types (ST) of *M. plutonius* strains were determined by multilocus sequence typing (MLST) [26] according to the protocol of Haynes et al. [27]. The sequence type of each strain was determined *in silico* using the public available ST data from the PubMLST database (as of 10 August 2017; http://pubmlst.org/). The goeBURST algorithm as implemented in the program PHYLOViZ v2.0 [28] was used to calculate and visualize the minimum spanning tree (MST) composed of sequence types and clonal complexes (CC). Orthologous proteins were identified with the program Proteinortho v5.11 (parameters: identity cutoff 50%, coverage cutoff 50%, e-value cutoff for blastp 1 × 10^−5^) [29] by using the protein sequences deduced from the 14 *Melissococcus* genomes as input. For this purpose, cat_seq v0.1 and cds_extractor v0.6 were used [30]. Based on these data, presence and absence of orthologous groups were converted into a simple binary matrix, and a gene content tree was calculated via RAxML v8.1.3 [31] with 1000 bootstrap re-samplings and the GAMMA model of rate heterogeneity. For visualization, the Dendroscope software v3.2.1 [32] was used. Harvest v1.1.2 together with Parsnp v1.2 and Gingr v1.2 as part of the Harvest software suite [33] were used to perform core genome alignments, calculate genome phylogeny, and identify and visualize single nucleotide polymorphisms (SNPs) and short insertions and deletions (Indels). *M. plutonius* 49.3 was used as a reference strain. Gubbins was used to identify loci with high densities of SNPs [34] that may indicate horizontal gene transfer (HGT). Results of this analysis were also used to reconstruct genome phylogeny based on a maximum likelihood approach, as implemented in Gubbins [34]. Mugsy (v1.2.3) [35] was used to produce a whole genome alignment of all 14 strains used as input for Gubbins. The resulting tree and HGT events were visualized using phandango [36].

The Phage Search Tool (PHAST) [37] was used to determine prophage sequences within bacterial genomes. The GIPSy software v1.1.1 (http://www.bioinformatics.org/ftp/pub/gipsy/) was used to detect genomic islands. Additionally, a blastp (e-value: 1 × 10^−50^) search of the deduced protein sequences against the virulence factor database (VFDB) [38] was performed to detect putative virulence factors. The detection of putative bacteriocins was done via BAGEL3 [39], Bactibase (database as of 17 February 2015) [40], and IMG-ER [22]. The MEROPS database v9.12 [41] was used to detect proteolytic enzymes and their substrates. BRIG v0.95 [42] and easyFig v2.1 [43] were used to visualize whole genome and genome region comparisons, respectively.

### 2.5. cDNA Synthesis and Reverse Transcription PCR (RT-PCR)

Three 5th instar worker larvae (*Apis mellifera*) displaying EFB symptoms (*M. plutonius* strains B5, H6, L9 all belonging to ST7, have been isolated from one of these larvae) were sampled from the same colony in Lützelflüh, Switzerland, in June 2013. Furthermore, healthy honey bee 5th instar larvae (Lohne, Germany, August 2014) were used as a negative control. Sampled larvae were frozen in liquid nitrogen immediately after collection. Honey bee larvae were individually homogenized in 200 µL sterile TE buffer (10 mM Tris-HCl (pH 7.4), 1 mM EDTA) supplemented with 3 mg/mL lysozyme. The homogenate (50 µL) was used for a fast EFB confirmation test using the EFB Diagnostic Test kit (Vita Europe, Basingstoke, UK) and 10 µL was used for colony PCR with specific primer pairs targeting the 16S rRNA gene of *M. plutonius* [44] (Table S1). Subsequently, parallel isolation of total DNA and RNA was performed by using 140 µL homogenate and the DNeasy Blood and Tissue kit and RNeasy Mini kit supplemented with RNAprotect Bacteria Reagent as recommended by the manufacturer (Qiagen, Hilden, Germany). RNA extracts were treated with DNase I (Thermo Scientific, Germany) and purified with RNeasy MinElute CleanUp kit (Qiagen, Hilden, Germany). Complete removal of DNA was verified by a PCR reaction targeting the 16S rRNA gene using a specific primer pair (16S-08F/16S-1504R) (Table S1) and DreamTaq DNA Polymerase as recommended by the manufacturer (Thermo Scientific, Germany). Purified RNA was transcribed to single-strand cDNA (sscDNA) using the QuantiTect Reverse Transcription kit (Qiagen, Hilden, Germany). The resulting sscDNA was used directly for RT-PCRs of single *M. plutonius* genes. Transcription of *M. plutonius* specific genes in EFB-infected larvae was tested using RT-PCR (for primers details see Table S1). These included the putative virulence factors endo-alpha-*N*-acetylgalactosaminidase, enhancin, and a toxin. The transcription of the 16S rRNA gene, *rpoD* (RNA polymerase sigma factor), and *rho* (transcription termination factor) were used as positive controls. Genomic DNA of strain 49.3 was used as positive control and strain S1 as negative control as the toxin gene is not present in this strain (Figure S1). For amplification of the 16S rRNA gene, genomic DNA and cDNA from a healthy honey bee larva was used as a negative control. All PCRs were performed using the Bioxact kit as recommended by the manufacturer (Bioline, London, UK).

## 3. Results

### 3.1. Sequence Types of Melissococcus plutonius Isolates and Clonal Complex Association

**Figure 1:**
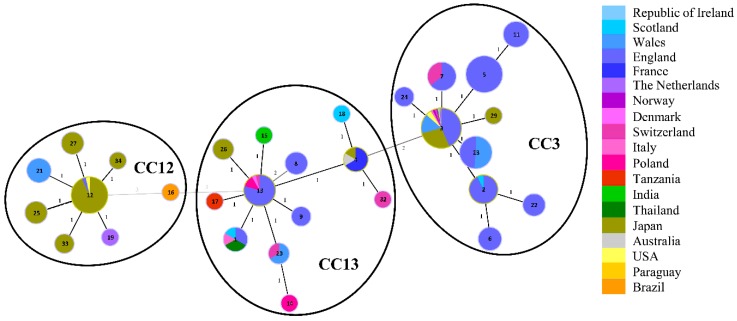
Minimum spanning tree of sequence types (STs) found in *Melissococcus plutonius* isolates from different countries. MLST data from this study were added to those reported previously [8,27,45] and resulted in the same three clonal complexes (CC3, CC12, and CC13) as reported previously. Altogether, 379 isolates were used to generate this tree using the PubMLST website (https://pubmlst.org/); for more details see Material and Methods—Genome analyses. Each circle represents a distinct ST – indicated by the different numbers in each circle, and the size indicates the frequency of occurrence. Closest relatives are linked with lines including distance labels. Black lines indicate a single allelic variant between STs and gray lines variation of at least two loci. Colors within circles represent the proportion of isolates of a particular ST that were found in the countries indicated on the right. The obtained data were submitted to PubMLST.

In order to analyze the molecular epidemiology and population structure of *M. plutonius* and unravel the phylogenetic relationship of 12 isolated strains from Norway and Switzerland, STs were assigned to all isolated strains. Based on the STs, CC were mainly calculated as single locus variants with some exceptions (Figure 1). Four isolates belong to ST3 (49.3, S1, 764-5B, and 765-6B) and five isolates to ST7 (21.1, 60, B5, H6, and L9). Both STs grouped into CC3 (Figure 1, Table 1 and Table S2). The remaining *M. plutonius* strains 119 and 90.0 belong to ST20 and ST13, respectively, and their corresponding STs are part of CC13 (Figure 1, Table 1 and Table S2). Interestingly, the ST profile from *M. plutonius* 82, another isolate from Switzerland, with *argE* 1, *galK* 9, *gbpB* 2, and *purR* 4, could not be assigned to an already existing ST. Thus, a novel ST (ST32) was defined within CC13. No strain could be allocated to the known CC12 to which the atypical strains (including DAT561) from Japan belong. The type strain *M. plutonius* ATCC 35311 from England was assigned to ST1 (CC13) according to Haynes et al. [27].

### 3.2. Genome Analysis—General Properties

The genomes of the typical strains *M. plutonius* 21.1, 49.3, 60, 82, 90.0, 119, B5, H6, L9, S1, 764-5B, and 765-6B range from 2.021 to 2.101 Mbp (2.062 ± 0.0203 Mpb, mean ± standard deviation (SD)) and comprise between 1589 and 1686 predicted protein-encoding genes (Table 1 and Table S2). For comparison, the genome sequences of the already known typical strain *M. plutonius* ATCC 35311 and atypical strain DAT561 were analyzed in the same way. To verify completeness and comparability of the analyzed genomes, we used checkM [25]. CheckM compared 542 markers across all genomes and showed that only 16-18 *Enterococcaceae* specific-marker are missing for each *M. plutonius* strain (Table S3). The 14 *Melissococcus* strains share a genome completeness of 93.88% ± 0.22% (mean ± SD), and strain heterogeneity and contamination were estimated as 0%. (Table S3). This shows that genomic variance across strains is very low. *M. plutonius* strains ATCC 35311, DAT561 and S1 have closed genomes without any gaps. Even these strains have the same completeness and contamination value as all other strains. From these results, we can conclude that missing genes (not identified using the same bioinformatical approach) in some strains, but detected in others, can be assigned as absent. As mentioned previously (Material and Methods) only sequences below 0.5 kbp have not been included in estimating genome sizes and for virulence gene screening. Hence, with such high values for genome completeness, it is very unlikely that genes have not been detected, caused by missing genome sequences. Comparing the different *Melissococcus* genomes with respect to their coding density revealed a high density with neglectable variance for the 14 different strains (79.72 ± 0.29%, mean ± SD), suggesting no substantial assembly or gene calling errors (Table S3).

**Figure 2:**
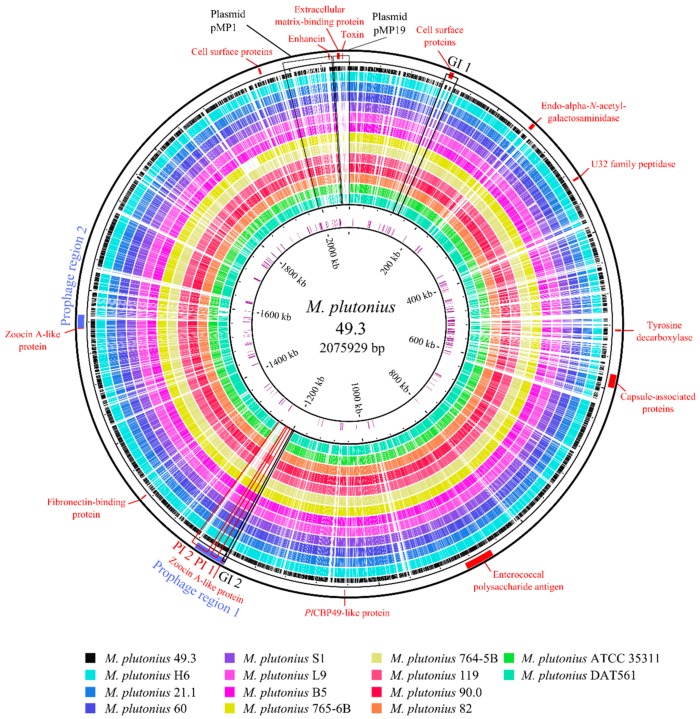
Circular genome map of *M. plutonius* 49.3. Comparison of the *M. plutonius* 49.3 genome to the genomes of strains H6, 21.1, 60, S1, L9, B5, 765-6B, 764-5B, 119, 90.0, 82, ATCC 35311, and DAT561 using the BRIG software [42]. The inner circle shows the positions of pseudogenes in the *M. plutonius* 49.3 genome, while virulence factors and prophage regions are depicted on the outer circle and are marked with red and blue blocks, respectively. Furthermore, pathogenicity (PI) and genomic islands (GI) are encircled and numbered in red and black. The plasmids pMP1 and pMP19 of *M. plutonius* 49.3 are indicated as well.

**Figure 3:**
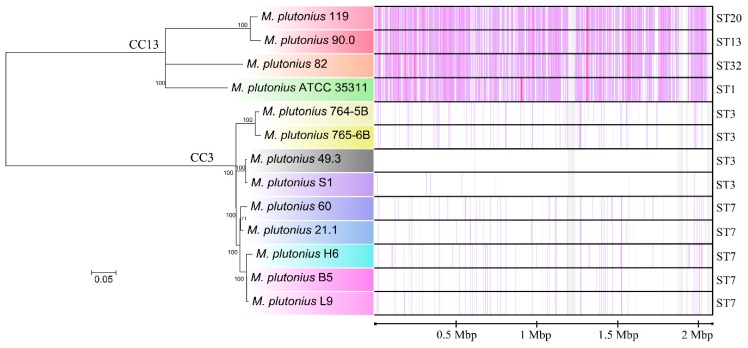
Phylogenetic tree based on core genome single nucleotide polymorphism (SNP)-typing of typical *M. plutonius* strains. The phylogenetic tree was obtained via the Harvest software suite [33]. *M. plutonius* 49.3 was set as the reference strain. The strains are marked in the same color code used in Figure 2. The SNP and Indel positions, in relation to the reference, are shown on the right hand side as violet lines. Sequence types are shown as well for comparison purposes.

Gene content comparisons were performed by using the genome of *M. plutonius* 49.3 as a reference (Figure 2). In general, the genomes are very similar in their gene content, except for a 19.4 kbp plasmid (pMP19) that is present only in 4 isolated strains (49.3, 21.1, 60 and H6). Based on these results, a phylogenetic tree was obtained via the Harvest software suite [33] through a core-genome alignment with SNP detection (Figure 3). This phylogeny resolves the relationship of the strains in more detail than MLST analysis. It confirms the close relationship between ST3 and ST7 strains within CC3, and shows that *M. plutonius* 82 is a sister taxon of *M. plutonius* ATCC 35311 within the monophyletic lineage of CC13 strains. The SNP based ‘Harvest’ phylogenetic tree was not corrected for recombination. Reanalyzing all genomes using Gubbins included the search for loci with signs of horizontal gene transfer. Comparing both phylogenies (Figure 3 and Figure S2) revealed that there is no difference between both trees. In other words, we did not detect relevant signs of recombination and horizontal gene transfer events in the phylogeny of our strains.

Interestingly, a high amount of pseudogenes was detected in all strains due to frame shifts and premature stop codons (75 to 156) (Table 1 and Table S2), which were caused by mutational events in coding regions like SNPs and Indels [46].

All strains harbor a plasmid with high DNA sequence similarity to the recently published plasmid pMP1 (NC_015517) of *M. plutonius* ATCC 35311 [11]. Moreover, for several strains such as 21.1, 49.3, 60, B5, and H6, it is indicated that they harbor additional plasmids. Strain B5 harbors a 42.7 kbp plasmid (pMP43) encoding phage proteins, which are also present in the chromosome of H6, L9, 49.3, S1, 60, 21.1, 764-5B, and 765-6B (see prophage region 1, Figure 2).

Based on the identification of orthologous proteins we calculated the core genome of this set of *M. plutonius* strains as 1304 proteins, which represents on average approx. 71% of the proteins encoded by a *M. plutonius* genome. The pan-genome of our strain panel comprises 1846 proteins.

Comparing the different genome properties, this is the first study providing evidence for multiple strain infections in a single honey bee larva. B5, H6 and L9 were isolated from the same larva but have different genome sizes, number of coding sequences and pseudogenes, and the pMP19 plasmid is only present in H6. All three strains cluster together closely (Figure 3), which might indicate that they are part of the local *M. plutonius* evolutionary diversity, rather than the result of independent infection events.

### 3.3. Genome Analysis—Detection of Putative Virulence Factors

**Figure 4:**
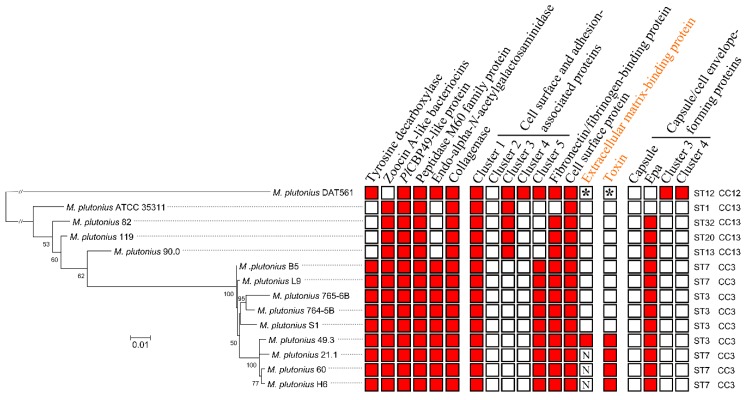
Gene content tree based on presence or absence of orthologous proteins. For constructing the phylogenetic tree, a presence/absence binary matrix was created from orthologous groups to calculate phylogeny with RAxML v8.1.3 [31]. The atypical strain *M. plutonius* DAT561 was used as the outgroup. Numbers at nodes are bootstrap values calculated from 1000 re-samplings to generate a majority consensus tree. The scale bar indicates divergence in presence or absence of proteins. STs are shown on the right. Color-filled boxes to the right of the organisms show the presence of the indicated proteins. Genes encoding putative virulence factors in red font are located on pMP19. An ‘N’ symbolizes that the respective open reading frame (ORF) is not complete due to a gap in the DNA sequence. An asterisk symbolizes that the respective ORF has been identified only in the second version of the DAT561 genome [47]. (Epa: enterococcal polysaccharide antigen).

Arai et al. [9] and Nakamura et al. [10] showed that one atypical strain DAT561 (CC12) was more virulent (laboratory infection assays) than two typical strains (DAT606-CC3, DAT585-CC13). Another recent study confirmed the high virulence of a CC3 strain and variable, mostly non-virulence, of CC13 strains [48]. However, data supporting general CC-specific differences in virulence are currently lacking. In order to look for gene patterns characteristic for each CC, which might show if virulence differences co-segregate with the three described CCs, we calculated a gene content tree based on the presence and absence of proteins in each strain (Figure 4). The gene content tree showed high similarity to the SNP-based phylogeny (Figure 3). The ST3/ST7 (CC3) strains cluster together as well as STs that belong to CC13 (ST1, 13, 20 and 32). Interestingly, the typical strains built different phylogenetic clusters (Figure 3). 

Atypical and typical strains might differ phenotypically under specific field conditions [9], and different regulation mechanisms for virulence were suggested [45]. To reveal putative differences at the genomic level, orthologous proteins in all strains were analyzed in detail using bioinformatical tools. We found 132 proteins, which are present in the atypical strain but were not identified in all typical strains (Data S2-Sheet 1a, Figure 4). Some of these potentially represent virulence factors or variations in metabolic properties necessary for a pathogenic lifestyle. The typical *M. plutonius* strains have 275 orthologues in common, which were not identified in the atypical strain DAT561 (Data S2-Sheet 1b). The majority of these orthologues are hypothetical or phage-related proteins, but several putative virulence factors were identified. In addition, putative virulence factors were determined by identifying genomic (GI 1, 2) and pathogenicity islands (PI 1, 2) and their associated virulence determinants (Data S2-Sheet 2, 3a, 3b). A summary of all identified putative virulence factors is depicted in Figure 4 and Data S2-Sheet 4, including bacteriocins, a tyrosine decarboxylase, *Pl*CBP49-like protein, enhancin, a collagenase, bacteria cell surface- and host cell adhesion-associated proteins, capsule and antigen-forming proteins, and a toxin.

### 3.4. Characterization of Genes Putatively Important for Melissococcus plutonius Survival and Pathogenicity

#### 3.4.1. Bacteriocins

A high number of bacteria produce peptides called bacteriocins, which possess antimicrobial activities against very closely related species or even against strains of the same species [49]. Seven (typical strains) or five (atypical strain DAT561) genes and gene clusters encoding putative bacteriocin biosynthesis and transport functions were identified in the *M. plutonius* genomes (Data S2-Sheet 4), sharing high similarity with putative bacteriocin biosynthesis clusters of *Enterococcus* and transport clusters of *Streptococcus* spp. (Figure S3). Two putative functional ORFs share low similarity with Zoocin A-like bacteriocins (Data S2-Sheet 4, see ‘Bacteriocin-associated proteins’, ORF1 and 2), and one with an unclassified bacteriocin determined by BAGEL3 [39] (Data S2-Sheet 4, see ‘Bacteriocin-associated proteins’, ORF3). Remarkably, ORF1 is only present in typical strains ST3 and ST7. On the contrary, ORF3 was found in all other STs determined in this study, excluding the atypical strain. In addition, we found lysozyme subfamily 2 domain/GH73 family domain-containing proteins (Data S2-Sheet 4), which might be involved in bacterial cell wall degradation [50].

#### 3.4.2. Tyramine

We identified an *Enterococcus*-type tyrosine decarboxylase gene cluster, which is involved in tyramine production (catalyzing the reaction from L-tyrosine to tyramine and CO_2_) [51]. The genes encoding the tyrosine decarboxylase of the typical strains 82, 90.0, 119 and ATCC 35311 are putatively non-functional due to a nonsense mutation (Data S2-Sheet 4). Kanbar et al. [52] showed that tyramine production of *Enterococcus faecalis* has highly toxic effects on honey bee larvae leading to classical EFB symptoms [53]. 

#### 3.4.3. Larval Glycoprotein and Peritrophic Matrix-Degrading Enzymes

**Figure 5:**
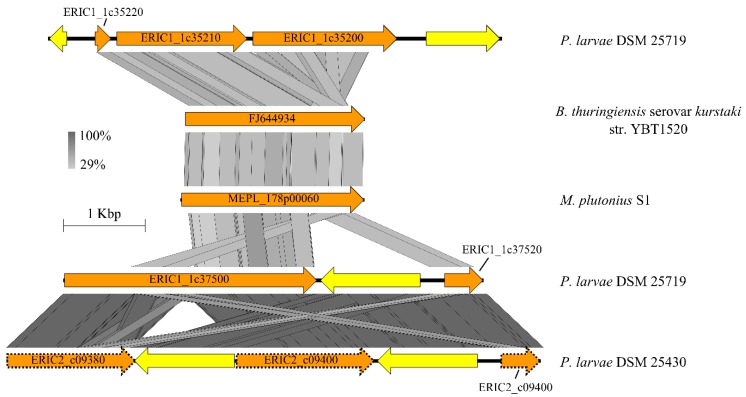
Comparison of the enhancin gene cluster of *M. plutonius* S1 with *P. larvae* DSM 25719, *Paenibacillus larvae* DSM 25430 and *Bacillus thuringiensis* serovar *kurstaki* str. YBT1520. The graphical presentation was done with the Easyfig software (minimum blast hit length of 50 bp) [43]. ORFs depicted as dotted arrows represent pseudogenes. ORFs related to enhancin are orange, and transposases are shown in yellow.

The peritrophic matrix lines the midgut of invertebrates and is comprised of secreted chitin and (glyco)proteins, mainly peritrophins [54]. In the *M. plutonius* genomes, a potential chitin-binding domain-containing protein, consisting of a signal peptide and a type 3 chitin-binding domain, was identified (Data S2-Sheet 4). It belongs to the ‘auxiliary activity family 10’ enzyme complex, a family of lytic polysaccharide monooxygenases, and exhibited 37% amino acid sequence similarity to *Pl*CBP49 (JX185746) of *Paenibacillus larvae*. *Pl*CBP49 is able to degrade the peritrophic matrix of the honey bee larva [55]. Additionally, a peptidase M60 family protein (enhancin), which can potentially degrade the peritrophic matrix of the honey bee larvae [56,57,58,59], was present in all strains (Figure 4 and Figure 5, Data S2-Sheet 4). It contains a signal peptide and shows high similarity to an enhancin-like protein of *Bacillus thuringiensis* serovar *kurstaki* str. (Figure 5). The peptidase M60 family protein also shows low amino acid sequence similarity to an M60 family protein of *P. larvae* DSM 25719 (22% identity with ERIC1_1c29890) [60] and it is homologous to several pseudogenes of *P. larvae* DSM 25719/25430, which are fragmented by transposase insertions or mutations (Figure 5) and are putatively non-functional. The typical *M. plutonius* strains harbor an identical enhancin protein (744 amino acids), whereas the enhancin of atypical strain DAT561 is slightly truncated (728 amino acids). However, analyzing the most recent version of the DAT561 genome [47] revealed that enhancin is not truncated and might be fully functional like the gene identified for the typical strains. Furthermore, we detected a gene encoding putative endo-alpha-*N*-acetylgalactosaminidase (EC 3.2.1.97) that catalyzes the release of oligosaccharides via hydrolysis of the *O*-glycosidic bond between alpha-acetylgalactosamine at the reducing end of mucin-type sugar chains (*O*-glycan) and serine/threonine residues of proteins, which is putatively non-functional in the *M. plutonius* strains 82, 90.0, 119 and ATCC 35311 due to nonsense mutations. 

To confirm expression of these potential virulence factors in larvae, we conducted a gene expression analysis using naturally infected honey bee larvae, which showed that enhancin as well as endo-alpha-*N*-acetylgalactosaminidase were transcribed in vivo during EFB pathogenesis (Figure S1), while an expression was not detected in a healthy honey bee larva (Figure S1).

#### 3.4.4. Bacterial Cell Curface- and Host Cell adhesion-associated Proteins

**Figure 6:**
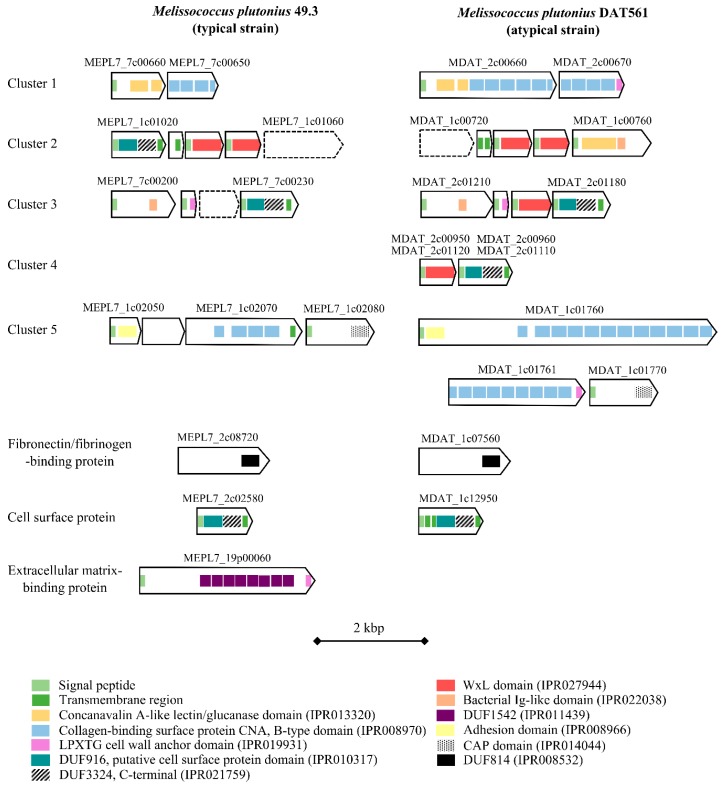
Domain structure of putative bacteria cell surface- and host cell adhesion-associated proteins with a putative role as virulence factors identified in *M. plutonius* 49.3 and DAT561. Signal peptides, transmembrane regions and domains were determined using InterProScan 5 [21], and are depicted using the color code shown in the legend. Cluster sizes range from 2 to 5.3 kbp in *M. plutonius* 49.3 and 1.7 kbp to 9.8 kbp in *M. plutonius* DAT561. The presence of orthologous genes and gene cluster identified in the other strains is shown in Figure 4 and Data S2-Sheet 4.

Altogether, five gene clusters and three single ORFs were associated with bacterial cell surface and bacteria adhesion to host matrix. Each typical strain has nonsense mutations in at least one of the cluster involved in adhesion. An overview of the identified bacteria cell surface- and host cell adhesion-associated proteins including their domain structures is depicted in Figure 6, and the presence and absence of selected proteins is shown in Figure 4 and Data S2-Sheet 4. The genomes of the typical strains encode less potentially functional bacteria cell surface- and host cell adhesion-associated proteins than the atypical strain DAT561. Two gene clusters (one and five) of the typical strains are putative remnants of clusters detected in the atypical strain DAT561. Cluster three contains one ORF with a nonsense mutation, and cluster four is missing in all typical strains (Figure 4).

A fibronectin/fibrinogen-binding domain (DUF (Domain of unknown function) 814)-containing protein was discovered in all strains examined in this study (Figure 4 and Figure 6). The corresponding ORF encodes a protein, which shares high similarity (70% identity) to the fibronectin-binding protein of *Enterococcus caccae* and *Enterococcus moraviensis* (WP_010772361 and WP_010765067).

A putative extracellular matrix-binding protein (MEPL7_19p00060, Figure 4 and Figure 6) is plasmid-encoded (pMP19) and is only present in the typical strains 21.1, 49.3, 60 and H6 (Data S2-Sheet 4). It contains eight copies of a DUF1542 domain. In *Staphylococcus aureus*, it was shown that some DUF1542-containing proteins are involved in cell cluster formation, cellular adhesion and antibiotic resistance [61,62]. 

#### 3.4.5. Toxin

Only the genomes of the typical *M. plutonius* strains 21.1, 49.3, 60 and H6 harbor a putative toxin-encoding ORF (Figure 4, Data S2-Sheet 4), while all other typical strains and the atypical strain DAT561 lack such a gene. However, this has to be revised for the atypical strain DAT561, as the latest version of this genome included the pMP19 plasmid [47]. The toxin, we designated ‘melissotoxin A’, is plasmid-encoded (pMP19). It shows 33% amino acid sequence identity to an epsilon toxin ETX/mosquitocidal toxin MTX2 family protein of *Brevibacillus laterosporus* (WP_018669999), which is a common secondary invader in EFB disease [63]. Most importantly, the melissotoxin A-encoding gene is expressed during infection in vivo (Figure S1). 

#### 3.4.6. Capsule and Cell Envelope-Forming Proteins

Capsules are a layer of surface-associated polysaccharides protecting bacteria against desiccation, attack from phages, antimicrobial peptides, and phagocytosis [64,65]. We detected four gene clusters, which are associated with capsule and cell envelope-forming proteins (Figure 4, Data S2-Sheet 4). Gene cluster 1 comprises a putative capsule locus, which was described for *E. faecium* strains by Palmer et al. [66]. The putative capsule-encoding gene clusters of *E. faecium* 504 and *E. caccae* ATCC BAA-1240 share high sequence similarity to this cluster, although all *Melissococcus* strains contain nonsense mutations in genes involved in capsule formation (Data S2-Sheet 4).

**Figure 7:**
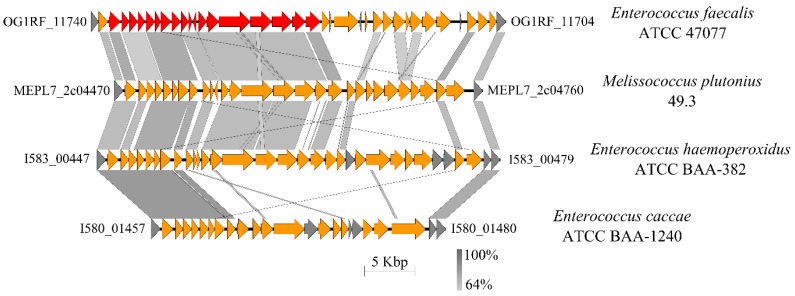
Comparison of a gene cluster of *M. plutonius* with gene clusters of *Enterococci* encoding Epa. ORFs labeled with locus tags represent the corresponding ends of the shown genome segments. ORFs related to the *epa*-locus are marked in orange, and ORFs encoding Epa of *Enterococcus faecalis* are depicted in red. Conserved hypothetical proteins are shown in gray. The gene cluster shows highest sequence similarity to Epa of *E. faecalis* and *Enterococcus haemoperoxidus*.

The second gene cluster exhibits a similar composition to the enterococcal polysaccharide antigen (*epa*)-locus of *E. faecalis* [67,68,69], *Enterococcus haemoperoxidus* and *E. caccae* (Figure 7). Epa is suggested as a virulence factor and facilitates resistance to bile salts and antimicrobial peptides [69,70]. *M. plutonius* ATCC 35311 and the atypical strain DAT561 are the only strains that have frameshift mutations in at least one gene of this cluster (Data S2-Sheet 4).

Clusters three and four consist of two ORFs each. Both are putatively only functional in the atypical strain DAT561 (Data S2-Sheet 4). ORFs belonging to these clusters encode lipid A-like transporters.

#### 3.4.7. Energy and Sugar Metabolism

**Figure 8:**
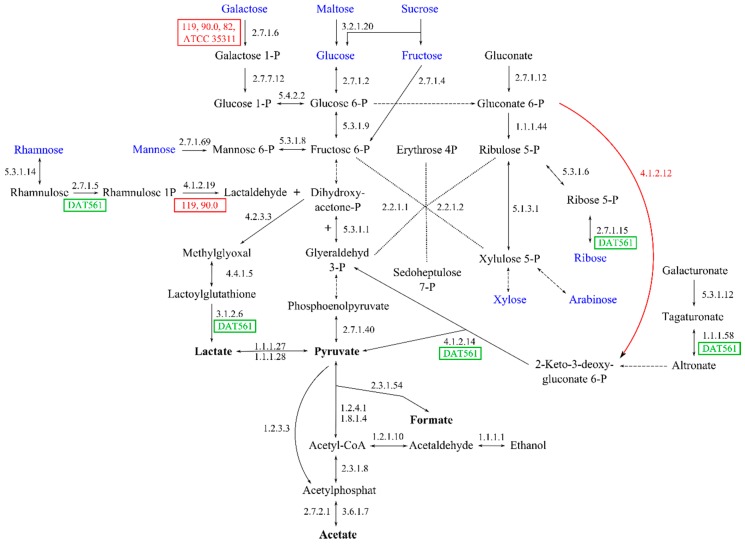
Genome-based analysis of glycolysis, pentose phosphate pathway, Entner-Doudoroff (ED) pathway, mixed acid fermentation and sugar interconversions (partly). The reactions are schematized (co-factors, co-substrates, CO_2_-formation are not shown). Dotted arrows indicate a summary of multiple reactions, which were found to be present in all strains. Gene products are visualized via EC numbers for the catalyzing enzymes. Green blocks indicate strain-specific reactions. Red blocks display all strains missing the respective enzyme. Sugars present in honey and degraded pectin backbones (Figure S3) are visualized in blue. Pyruvate and putative end products are shown in bold. All strains lack a pyruvate-decarboxylase (EC 4.1.1.1), which is part of ethanol fermentation. Additionally, all strains lack a phosphogluconate dehydrogenase (red arrow, EC 4.1.2.12), which is part of the Entner-Doudoroff-pathway.

Competition for resources with the host results in an evolutionary pressure on bacteria. Therefore, we studied in more detail the potential pathways for energy and sugar metabolism of *M. plutonius*. All *M. plutonius* strains lack a tricarboxylic acid (TCA) cycle and the electron transport system for oxidative phosphorylation. Enzymes for a glycolysis system were found in all strains, but the genes encoding pyruvate kinase and transketolase of the atypical strain DAT561 are interrupted by frameshift mutations in the original genome sequence of this strains [12]. A recently updated version shows that both genes are not interrupted [47]. Enzymes required for homolactic acid fermentation were identified, but a glucose-6-phosphate dehydrogenase, 6-phosphogluconolactonase, and a decarboxylating 6-phosphogluconate dehydrogenase as part of the heterolactic acid fermentation are also encoded. An overview of glycolysis, the pentose phosphate pathway, the Entner-Doudoroff pathway, mixed acid fermentation, sugar interconversions (partly) and pyruvate metabolism of *M. plutonius* is shown in Figure 8. Additionally, amino acid decarboxylation and the arginine deiminase pathway can contribute to energy production. Finally, we detected a number of genes encoding enzymes that target plant cell wall polysaccharides (e.g., of pollen and beebread) as described for the honey bee gut microbiota [71] (Figure S4).

## 4. Discussion

The focus of this study was the genome-based identification of genes putatively relevant for survival and virulence (pathogenicity) of the honey bee pathogen *M. plutonius*. We used the obtained bioinformatical results to discuss critical steps for the pathogenesis and infection of *M. plutonius*; including proliferation of *M. plutonius* in the gut of the honey bee larva, competition for host resources and putative encapsulation of *M. plutonius*. However, nearly all of the pathogenesis mechanisms are completely unknown and consequently purely speculative. *M. plutonius* may have a different lifestyle as genome data showed, with genes which might also be relevant for an invasive lifestyle and actively killing honey bee larvae. The following discussion is based on the pure presence of the identified genes in the *M. plutonius* genomes and experimental evidence is needed to develop a fully functional infection model. We did not include the potential function of the secondary invaders (*B. laterosporus*, *E. faecalis*, *Paenibacillus alvei*, and *Achromobacter eurydice* (but see [72] for the controversial position of *A. eurydice*)) that are usually found in the remains of EFB-diseased honey bee larvae. Our genome analyses revealed that all *M. plutonius* strains are putatively able to degrade the pectin backbone of the pollen cell wall using a large variety of enzymes. Interestingly, the strains differ in their genetic equipment of these enzymes, as different enzymes are putatively non-functional due to mutations in the corresponding genes of all typical strains from Norway and Switzerland (Figure S4). Pectin degradation might result in pollen perforation and therefore in the release of its nutrient-rich content [71]. All strains harbor genes encoding enzymes for the essential energy metabolism pathways glycolysis and the pentose phosphate pathway. The putative lack of function of transketolase and pyruvate kinase of *M. plutonius* DAT 561 could be a sequencing error of the 454 sequencing approach chosen by Okumura et al. [12], which is not suitable to dissolve homopolymer stretches [73]. With the second version of the genome, using a different sequencing approach, both genes are present without any lack of function [47]. As shown in Figure 8 and Figure S3, the atypical strain DAT561 is putatively able to use a variety of sugar substrates as energy and carbohydrate sources via glycolysis, the pentose phosphate pathway, ED-pathway and sugar interconversions, which supports recent results [9]. As these substrates are ingredients of honey, royal jelly, pollen and beebread, the atypical strain DAT561 is more adapted to the natural resources found in the larval gut than typical strains. This might be the reason for faster growth of the atypical strain DAT561 in artificially infected larvae and consequently its higher virulence [10]. 

Besides the metabolic differences between typical and atypical strains, the production of tyramine by *M. plutonius* might be toxic for honey bee larvae [52]. It was shown that the production of tyramine led to a classic EFB symptom, whereas tyramine-treated larvae changed their color to yellow/brown [52]. Interestingly, the typical strains 82, 90.0, 119 and ATCC 35311 (all belonging to CC13) lack the required tyrosine decarboxylase (Figure 4), which could lead to decreased virulence of this clonal complex, as shown recently [48]. Here, we speculate that the assimilation of food and putatively the production of tyramine by *M. plutonius* might be the first steps in EFB pathogenesis and impact the further development of the honey bee larva severely; however, experimental data are needed to confirm this assumption.

During the infection cycle it might be essential for *M. plutonius* to be able to compete with part of the natural microbiota of the honey bee gut system. In the genomes of our strain panel, we found three putative genes encoding bacteriocin (antimicrobial peptides) biosynthesis and only the genome of the atypical strain *M. plutonius* DAT561 lacks the respective genes (Figure 4, Data S2-Sheet 4). The ability to produce bacteriocins in the space-limited and nutrient-embattled environment of the larval gut might be an advantage, though the lack of the genes for strain DAT561 is an argument against bacteriocins being relevant for pathogenesis or virulence. 

Additionally, only the atypical strain DAT561 and the typical strain *M. plutonius* ATCC 35311 lack the complete gene cluster encoding the biosynthetic machinery for Epa (Figure 4 and Figure 7). Teng et al. [74] showed that the *epa* locus is involved in the biosynthesis of a rhamnopolysaccharide, which is important for biofilm formation and virulence in a mouse peritonitis model [70,74], but also facilitates resistance to antimicrobial peptides. If biofilm formation is necessary for growth and virulence of *M. plutonius* it might be realized via the biosynthetic machinery for Epa, at least for the typical strains. However, biofilm formation might not be prerequisite for increased or high virulence, as indicated by the lack in the DAT561 genome.

Virulence of pathogenic bacteria of insects is mostly determined by the ability to degrade host glycoproteins and metabolizing resulting carbohydrates. The honey bee larval gut is coated by a chitin-containing peritrophic matrix, which is degraded during *P. larvae* infection [75]. This ability to destroy the peritrophic matrix has so far not been described for *M. plutonius*. Takamatsu et al. [76] showed for the strain DAT561 that peritrophic membrane and midgut epithelial cells were disintegrated and partly absent in badly infected larvae, and only a few cells were detected outside the midgut lumen. The same study found evidence that *M. plutonius* secretes unknown molecules to infiltrate the peritrophic matrix [76]. Nevertheless, successful invasion and proliferation in the haemocoel has not been reported so far. We identified several peritrophic matrix degrading proteins (e.g., peptidase M60 family protein (enhancin), endo-alpha-*N*-acetylgalactosaminidase), proteins involved in adhesion to host extracellular matrix (e.g., putative collagen adhesins, S layer and cell surface proteins, and a fibronectin/fibrinogen-binding protein), proteases and proteolytic enzymes; however, functionality and relevance for pathogenicity of all these proteins have to be verified in vivo.

Regarding pathogenicity, a plasmid (pMP19) was found in *M. plutonius* 49.3 with highly similar contigs in strains 21.1, 60, and H6. The plasmid comprises 20 ORFs of which two ORFs encode for an extracellular matrix-binding protein and melissotoxin A. Interestingly, the virulence plasmid pMP19 is not stably maintained during in vitro propagation. The five-time laboratory-passage of *M. plutonius* 49.3 cured the strain from the plasmid and resulted in strain *M. plutonius* S1. Furthermore, *M. plutonius* B5, H6 and L9 were all isolated from the same EFB-infected honey bee larva and exhibited a close phylogenetic relationship (Figure 3), but only strain (H6) still harbored the plasmid after three cultivation steps. It is already known that typical strains of *M. plutonius* lose their pathogenicity after several cultivation steps in the laboratory [9], which is most likely due to the loss of plasmid pMP19. We hypothesize that the typical strains 21.1, 49.3, 60 and H6 might be more virulent than the other typical strains analyzed in this study due to the presence of the putative virulence plasmid pMP19. Nevertheless, other genetic determinants must still be important for virulence, because the atypical strain DAT561 remains virulent even after multiple cultivation steps [9,10] and lacks pMP19 in the original genome sequence. However, a very recent sequencing effort, to increase coverage and sequence verification of strain DAT561, revealed that plasmid pMP19 is present in this strain [47] and might therefore be relevant for its high virulence. The extracellular matrix-binding protein and melissotoxin A are both present on DAT561 plasmid pMP19, may expressing the same function as discussed above for the typical strains. A plausible reason for the non-identification of the pMP19 plasmid and genes identified with frame-shifts in our and Okumura’s previous sequence analysis [12] might be the previous sequencing approach that may have caused more sequencing errors. The plasmid was also identified in several other, but not all, CC3, CC12 and CC13 strains [77].

If nutrients are depleted, several Gram-positive bacteria undergo sporulation. However, there are only a few historic references describing that *M. plutonius* forms a capsule [78,79] that allows survival in feces and wax for several months up to several years [79]. Other than that, no evidence is available that *M. plutonius* produces capsules in vivo in infected larvae, and at least for DAT561, capsule-formation has not been detected using light and electron microcopy [76]. This is well in line with our bioinformatical results, as we detected a gene cluster encoding capsule-forming proteins in all *M. plutonius* strains analyzed in this study (Data S2-Sheet 4), with nonsense mutations in at least one gene needed for its biosynthesis (Figure 4) for all strains. Thus, capsule-formation in *M. plutonius* is partly inhibited or completely lost due to mutations of capsule-associated genes.

In the last decade, genome qualities and quantities became high-grade on a nearly yearly basis with the development of new deep sequencing technologies. Resequencing genomes with higher coverage and including genomes of other subspecies, genotypes or strains improves genome quality significantly. The current study was based on 12 newly sequenced typical strains, the genome of the reference strain ATCC 35311 [11] and the original genome of the atypical strain DAT561 [12]. By having a look in the second version of the DAT561 genome [47], it became clear that sequencing errors have been reduced drastically. Comparing the original and newly available genome we could see that enhancin is no longer truncated and has the same size as for the typical strains, and atypical strain genes encoding pyruvate kinase and transketolase are not interrupted by frameshift mutations and are probably fully functional.

## 5. Conclusions

Based on the identification of putative virulence genes from different *M. plutonius* genomes, future EFB research can now study the infection in more detail to develop a pathogenesis model. The genetic equipment coding for virulence factors differs between most strains (Figure 4). Typical and atypical strains share a *Pl*CBP49-like protein, enhancin, collagenase and bacterial cell surface proteins, which putatively represent basic virulence factors of *M. plutonius* strains that are needed for infection of honey bee larva. Moreover, we hypothesize differences in virulence within the typical strains, as the typical strains belonging to CC13 (*M. plutonius* ATCC 35311, 82, 90.0 and 119) lack putatively important virulence factors (e.g., tyrosine decarboxylase, endo-alpha-*N*-acetylgalactosaminidase) in comparison to CC3 (ST3/7). Additionally, strains harboring the virulence plasmid pMP19 may have additional advantages versus strains lacking the plasmid. Typical and atypical strains most likely have different virulence mechanisms, with atypical strain DAT561 encoding for different bacteria cell envelope-associated and host cell adhesion-associated proteins. However, the atypical strain might compensate this with faster growth in the larval gut by increased metabolic capabilities with respect to usage of different nutrient sources. Faster nutrient consumption of atypical strains lead to starvation of the honey bee larvae. We assume that in case of atypical strains the combination of fast nutrient consumption and establishment of virulence factors leads to an accelerated death of the honey bee larvae. Nevertheless, additional infection studies are needed to predict a difference in virulence between strains and clonal complexes. Such studies also have to be tested with different host genetic backgrounds, as host genotype contributes to the course of the disease [48]. Therefore, phenotypic tests with isogenic mutant strains will be needed to evaluate their virulence properties in artificially infected honey bee larvae.

## Figures and Tables

**Table 1:** General data of *M. plutonius* strains used in this study.

Strain	Origin ^1^	ST (CC) ^2^	Classification	Genome Size [Mbp]	CDS ^3^	Pseudogenes	Plasmids
**49.3**	CH	ST3 (CC3)	Typical strain	2.076	1638	140	pMP1, pMP19
**S1**	CH	ST3 (CC3)	Typical strain	2.074	1609	151	pMP1
**21.1**	CH	ST7 (CC3)	Typical strain	2.077	1629	145	pMP1, pMP19
**60**	CH	ST7 (CC3)	Typical strain	2.072	1633	143	pMP1, pMP19
**B5**	CH	ST7 (CC3)	Typical strain	2.101	1686	146	pMP1, pMP43
**H6**	CH	ST7 (CC3)	Typical strain	2.075	1633	145	pMP1, pMP19
**L9**	CH	ST7 (CC3)	Typical strain	2.059	1616	145	pMP1
**82**	CH	ST32 (CC13)	Typical strain	2.048	1614	129	pMP1
**90.0**	CH	ST13 (CC13)	Typical strain	2.067	1642	131	pMP1
**119**	CH	ST20 (CC13)	Typical strain	2.040	1614	127	pMP1
**764-5B**	NO	ST3 (CC3)	Typical strain	2.046	1605	145	pMP1
**765-6B**	NO	ST3 (CC3)	Typical strain	2.021	1589	142	pMP1
**ATCC 35311**	GB-ENG	ST1 (CC13)	Typical strain	2.069	1594	156	pMP1
**DAT561**	JP	ST12 (CC12)	Atypical strain	2.045	1595	75	pMP1, pMP19 ^4^

^1^ CH: Switzerland, NO: Norway, GB-ENG: England, JP: Japan; ^2^ Sequence type (ST) and clonal complex (CC); ^3^ CDS stands for coding sequences; ^4^ recently identified in the new version of the genome [47].

## Supplementary Materials

The following are available online at http://www.mdpi.com/2073-4425/9/8/419/s1, Data S1: GenBank files of *M. plutonius* ATCC 35311 and DAT561, Data S2: Detection of putative virulence factors, Figure S1: Expression of *M. plutonius* putative virulence factors during infection, Figure S2: Phandango visualization of Mugsy phylogenetic tree with Gubbins horizontal gene transfer (HGT) events, Figure S3: Comparison of bacteriocin biosynthesis and transport clusters of *M. plutonius* S1 with clusters of *M. plutonius* DAT561, *E. faecalis* FLY1 (accession no. NZ_ACAR00000000) and *Streptococcus iniae* ISNO (accession no. CP007587), Figure S4: Pectin degradation by *M. plutonius*, Table S1: Primer used in this study, Table S2: General data of *M. plutonius* strains used in this study, Table S3. CheckM results for *M. plutonius* genome completeness, based on the marker set for *Enterococcaceae* (with 55 genomes).
