# Supplementary material for: Comparative Genomics and Description of Putative Virulence Factors of Melissococcus plutonius, the Causative Agent of European Foulbrood Disease in Honey Bees
Source: Genes (Basel). 2018 Aug 20;9(8):419. doi: 10.3390/genes9080419 (PMC6116112; doi:10.3390/genes9080419)
Supplement: Supplementary file 1 [file genes-09-00419-s001.zip › Supplementary material-Figures and Tables.docx]

Supplementary material

Comparative genomics and description of putative virulence factors of *Melissococcus plutonius*, the causative agent of European foulbrood disease in honey bees

Marvin Djukic ^1^, Silvio Erler ^2^, Andreas Leimbach ^1^, Daniela Grossar ^3,4^, Jean-Daniel Charrière ^3^, Laurent Gauthier ^3^, Denise Hartken ^1^, Sascha Dietrich ^1^, Heiko Nacke ^1^, Rolf Daniel ^1^ and Anja Poehlein ^1,^*

^1^ Department of Genomic and Applied Microbiology & Göttingen Genomics Laboratory, Institute of Microbiology and Genetics, Georg-August-University of Göttingen, 37077 Göttingen, Germany; mdjukic@posteo.de, aleimba@gwdg.de, denise.hartken@uni-goettingen.de, hnacke@gwdg.de, rdaniel@gwdg.de, apoehle3@gwdg.de

^2^ Molecular Ecology, Institute of Biology, Martin-Luther-University Halle-Wittenberg, Hoher Weg 4, 06099 Halle (Saale), Germany; erler.silvio@gmail.com

^3^ Swiss Bee Research Center, Agroscope, 3003 Bern, Switzerland; daniela.grossar@gmail.com, jean-daniel.charriere@agroscope.admin.ch, ruchersastet@club.fr,

^4^ Department of Ecology and Evolution, Biophore, UNIL-Sorge, University of Lausanne, 1015 Lausanne, Switzerland; daniela.grossar@gmail.com

***** Correspondence: apoehle3@gwdg.de; Tel.: +49-551-3933655


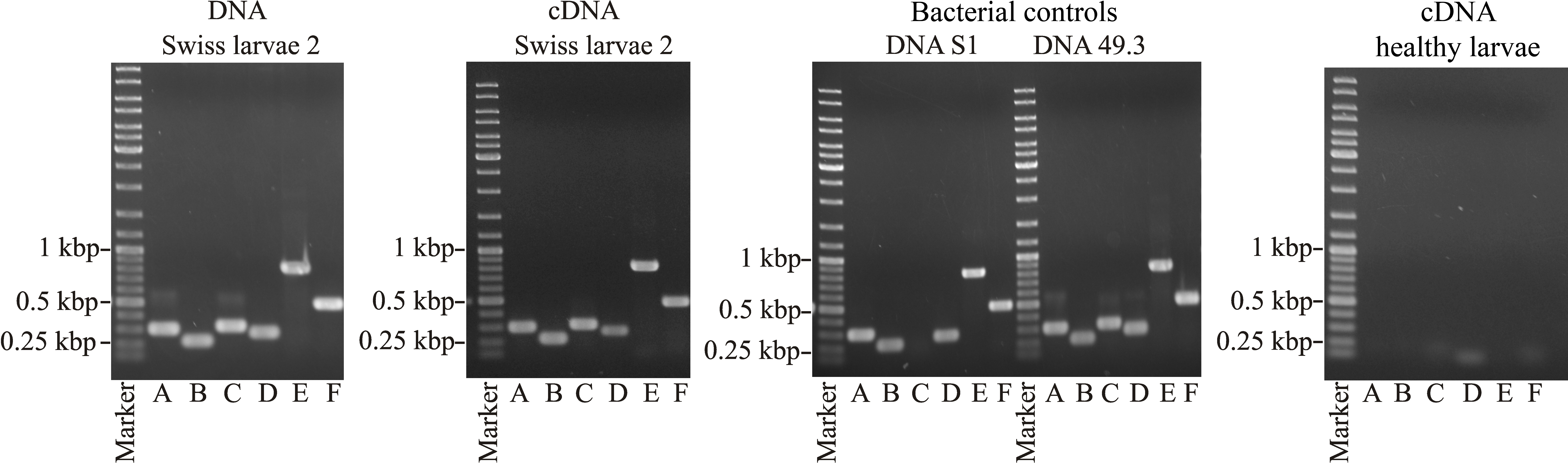


**Figure S1: Expression of *M. plutonius* putative virulence factors during infection.** On the basis of isolated DNA and cDNA from EFB-infected (Swiss larva) and healthy larvae (German larvae), we tested the presence and expression of A - endo-alpha-*N*-acetylgalactosaminidase, B - enhancin, C - toxin, D - transcription termination factor *rho*, E - 16S rRNA, F - RNA polymerase sigma factor *rpoD* and the 16S rRNA gene of *M. plutonius* via RT-PCR. In all EFB-infected larvae positive PCR products were obtained for DNA and cDNA. Further, we used genomic DNA of *M. plutonius* strain S1 and 49.3 as positive control for our marker genes, and as negative control for the toxin gene.


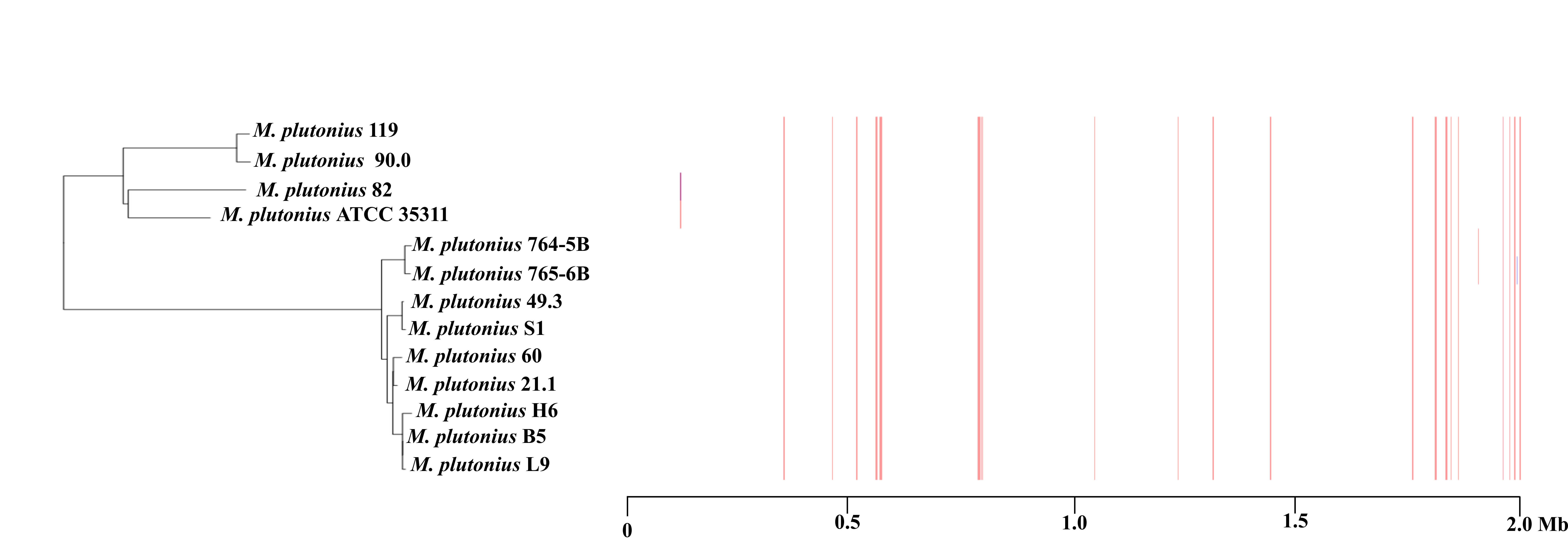


**Figure S2:** **Phandango visualization of Mugsy phylogenetic tree with Gubbins horizontal gene transfer (HGT) events.** Red bars represent HGT events in relative position within the genomic background. Height of the red bars represent the strains in relation to the phylogenetic tree that share a specific HGT event.


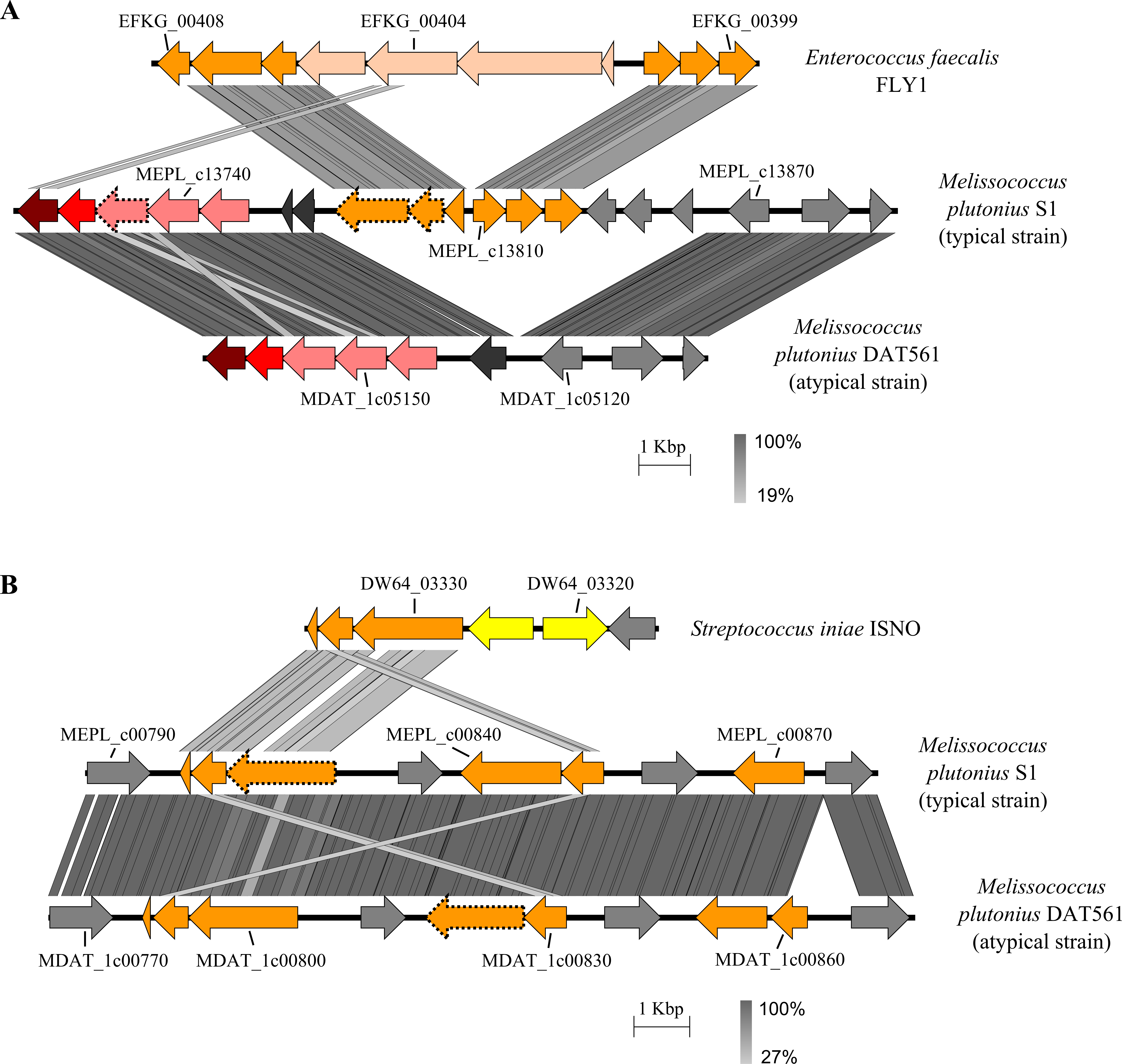


**Figure S3:** **Comparison of bacteriocin biosynthesis and transport clusters of *M. plutonius* S1 with clusters of *M. plutonius* DAT561, *E. faecalis* FLY1 (accession no. NZ_ACAR00000000) and *Streptococcus iniae* ISNO (accession no. CP007587).** The graphical presentation was done with the Easyfig software (minimum blast hit length of 50 bp) [43]. ORFs related to bacteriocin biosynthesis and transport are orange-shaded, an iron uptake system is red-shaded, transposases are yellow and genomic context is shown in gray and black. ORFs depicted as dotted arrows represent pseudogenes. The bacteriocin biosynthesis cluster one (A) shows high similarity to a cluster present in *E. faecalis* FLY1, *M. plutonius* DAT561 lacks this region completely. Cluster two (B) represents a bacteriocin transport cluster. Three ORFs of cluster 2 share similarity to ORFs present in *S. iniae* ISNO. *M. plutonius* DAT561 encodes an additional ORF (MDAT_1c00860), encoding a transcriptional regulatory protein. *M. plutonius* S1 is chosen as a representative for all typical strains in this study for this analysis.


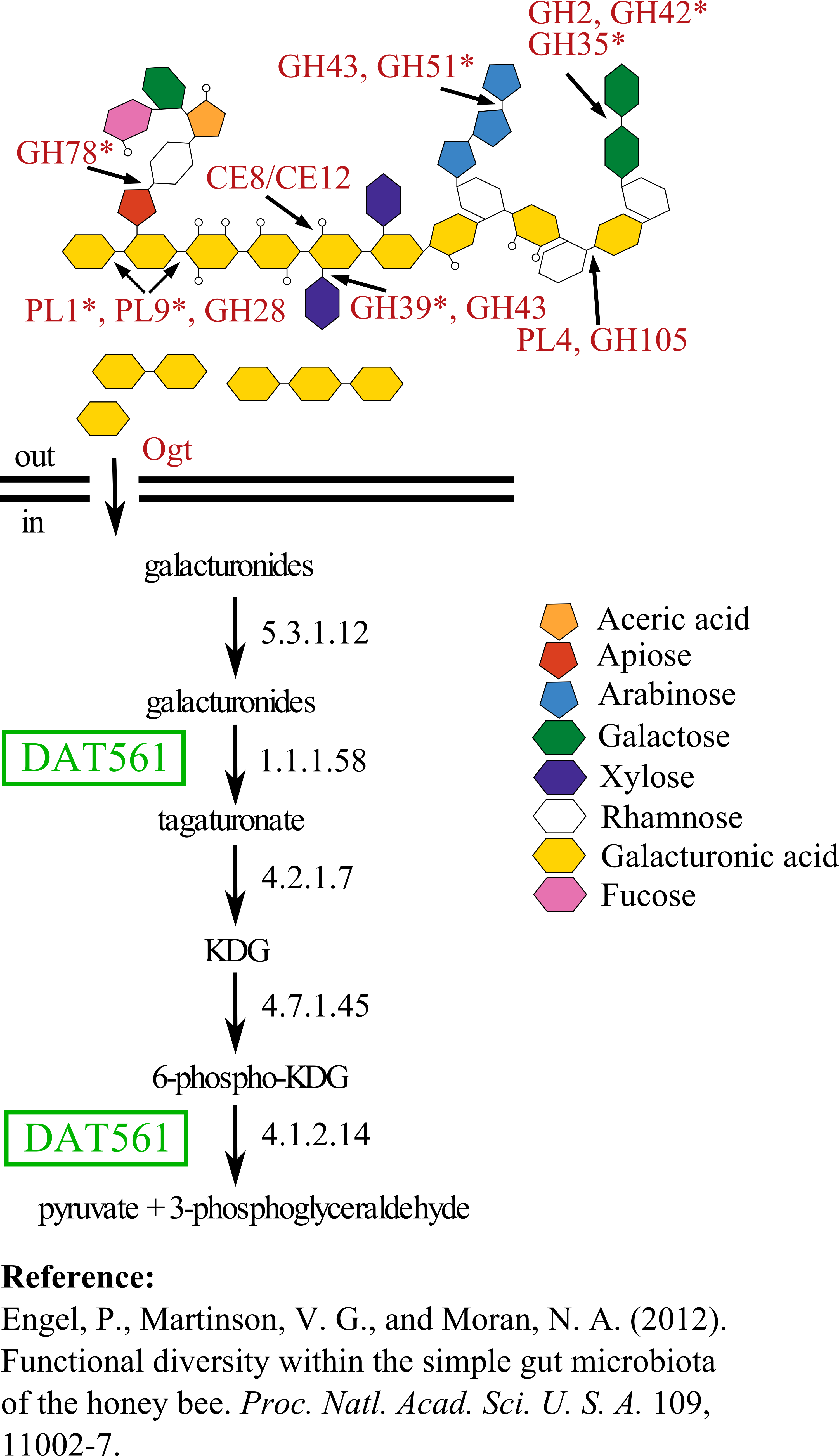


**Figure S4: Pectin degradation by *M. plutonius*.** Adaptation of Fig 3A from Engel et al. [71]. A number of genes encoding plant cell wall-degrading enzymes (including pollen walls) were identified in the genomes of all *M. plutonius* strains. Shown are identified families of glycoside hydrolases (GH), polysaccharide lyases (PL) and carbohydrate esterases (CE). Ogt is an acronym for oligogalacturonide transporter. Gene products are symbolized via EC numbers. Asterisks mark putative absence in at least one of the strains. Genes needed for the galacturonide conversion pathway are only completely present in *M. plutonius* DAT561, as genes encoding for tagaturonate reductase UxaB (EC 1.1.1.58) and 2-dehydro-3-deoxy-phosphogluconate aldolase KdgA (EC 4.1.2.14) are putatively dysfunctional in the typical strains. PL1 is missing in *M. plutonius* DAT561. PL9, GH35, and GH42 are present only in strains 119, 90.0, 82, ATCC 35311, and DAT561. Additionally, a GH78 was detected only in DAT561, an extracellular GH51 is missing in strain 765-6B, and a GH39 is present only in *M. plutonius S1* and ATCC 35311.

**Table S1. Primer used in this study.**

| **Target gene** | **Primer pairs used in this study** | **Sequence 5’-3’** | **Reference** | **Product size (approx.)** | **Used in Materials and Methods chapter** |
| --- | --- | --- | --- | --- | --- |
| 16S rRNA | 16S-08F | 5’-AGAGTTTGATCCTGGC-3’ | Kim *et al*. 2009, modified; | 1.5 kbp | cDNA synthesis |
|  | 16S-1504R | 5’-TACCTTGTTACGACTT-3’ | Baker *et. al*. 2003, modified |  |  |
| 16S rRNA | MEPL-16S_for | 5’-GAAGAGGAGTTAAAAGGCGC-3’ | Govan *et al*. 1998 | 0.83 kbp | RT-PCR |
|  | MEPL-16S_rev | 5’-TTATCTCTAAGGCGTTCAAAGG-3’ |  |  |  |
| RNA polymerase sigma factor | rpoD_MEPL_for | 5’-CCCGTGTGATCGCTTGTC-3’ | This study | 0.45 kbp | RT-PCR |
|  | rpoD_MEPL_rev | 5’-ACCTTTTACATTAAGTGCAGGTG-3’ |  |  |  |
| Transcription termination factor | rho_MEPL_for | 5’-TAACATCACCATTAACACTGCG-3’ | This study | 0.24 kbp | RT-PCR |
|  | rho_MEPL_rev | 5’-AACGCCTCTTTATCCCAATCG-3’ |  |  |  |
| Endo-alpha-*N*-acetylgalactosaminidase | endo_MEPL_for | 5’-AGTCACAGGTCAGGTAGAAGG-3’ | This study | 0.24 kbp | RT-PCR |
|  | endo_MEPL_rev | 5’-GGGATTTGAACGGTATAGGTAGC-3’ |  |  |  |
| Enhancin | enh_MEPL_for | 5’-TGTTTGGAGGTGCTTATCAGG-3’ | This study | 0.16 kbp | RT-PCR |
|  | enh_MEPL_rev | 5’-ACAATCTCACCGTCAATTTTTCC-3’ |  |  |  |
| Melissotoxin A | tox_MEPL_for | 5’-GCTCAAGCAGCAACTTTTACG-3’ | This study | 0.31 kbp | RT-PCR |
|  | tox_MEPL_rev | 5’-TTCCCCTGGTATTACTTGTAGATG-3’ |  |  |  |
| 16S rRNA | U341F | 5'-CCTACGGGRSGCAGCAG-3' | Baker *et al*. 2003; | 0.7 kbp | RT-PCR |
|  | 1061R | 5'-CRRCACGAGCTGACGAC-3' | Andersson *et al*. 2008 |  |  |

**References:**

Andersson, A. F., Lindberg, M., Jakobsson, H., Bäckhed, F., Nyrén, P., and Engstrand, L. (2008). Comparative analysis of human gut microbiota by barcoded pyrosequencing. *PLoS One* 3, e2836. doi:10.1371/journal.pone.0002836.

Baker, G. C., Smith, J. J., and Cowan, D. A. (2003). Review and re-analysis of domain-specific 16S primers. *J Microbiol Methods* 55, 541–555. doi:10.1016/j.mimet.2003.08.009.

Govan, V. A., Brözel, V., Allsopp, M. H., and Davison, S. (1998). A PCR detection method for rapid identification of *Melissococcus pluton* in honeybee larvae. *Appl Environ Microbiol* 64, 1983–1985.

Kim, Y. M., Ahn, C. K., Woo, S. H., Jung, G. Y., and Park, J. M. (2009). Synergic degradation of phenanthrene by consortia of newly isolated bacterial strains. *J Biotechnol* 144, 293–298. doi:10.1016/j.jbiotec.2009.09.021.

**Table S2.** **General data of *M. plutonius* strains used in this study.**

| **Strain** | **Origin** | **ST (CC)^a^** | **Classification** | **Genome contig count** | **Genome size [Mbp]** | **CDS^b^** | **Pseudo-genes** | **Plasmids** | **GenBank accession number** | **Coverage** | **Reference** |
| --- | --- | --- | --- | --- | --- | --- | --- | --- | --- | --- | --- |
| **49.3** | Switzerland | ST3 (CC3) | Typical strain | 9 | 2.076 | 1,638 | 140 | pMP1, pMP19 | JSBA00000000 | 102x | This study |
| **S1** | Switzerland | ST3 (CC3) | Typical strain | 2 | 2.074 | 1,609 | 151 | pMP1 | CP006683-CP006684 | 67x | This study |
| **21.1** | Switzerland | ST7 (CC3) | Typical strain | 8 | 2.077 | 1,629 | 145 | pMP1, pMP19 | JSAY00000000 | 338x | This study |
| **60** | Switzerland | ST7 (CC3) | Typical strain | 13 | 2.072 | 1,633 | 143 | pMP1, pMP19 | JSBE00000000 | 185x | This study |
| **B5** | Switzerland | ST7 (CC3) | Typical strain | 6 | 2.101 | 1,686 | 146 | pMP1, pMP43 | JSAW00000000 | 485x | This study |
| **H6** | Switzerland | ST7 (CC3) | Typical strain | 11 | 2.075 | 1,633 | 145 | pMP1, pMP19 | JSBC00000000 | 220x | This study |
| **L9** | Switzerland | ST7 (CC3) | Typical strain | 14 | 2.059 | 1,616 | 145 | pMP1 | JSBD00000000 | 240x | This study |
| **82** | Switzerland | ST32 (CC13) | Typical strain | 15 | 2.048 | 1,614 | 129 | pMP1 | JSBF00000000 | 202x | This study |
| **90.0** | Switzerland | ST13 (CC13) | Typical strain | 17 | 2.067 | 1,642 | 131 | pMP1 | JSAZ00000000 | 102x | This study |
| **119** | Switzerland | ST20 (CC13) | Typical strain | 15 | 2.040 | 1,614 | 127 | pMP1 | JSBB00000000 | 103x | This study |
| **764-5B** | Norway | ST3 (CC3) | Typical strain | 10 | 2.046 | 1,605 | 145 | pMP1 | JSAV00000000 | 1040x | This study |
| **765-6B** | Norway | ST3 (CC3) | Typical strain | 10 | 2.021 | 1,589 | 142 | pMP1 | JSAX00000000 | 652x | This study |
| **ATCC 35311** | England | ST1 (CC13) | Typical strain | 2 | 2.069 | 1,594 | 156 | pMP1 | Supplementary Data 1 (Original data: NC_015516.1 and NC_15517.1) | 44x | Okumura *et al*. 2011 |
| **DAT561** | Japan | ST12 (CC12) | Atypical strain | 2 | 2.045 | 1,595 | 75 | pMP1, pMP19 ^c^ | Supplementary Data 1 (Original data NC_016938 and NC_018265.1) | 50x | Okumura *et al*. 2012 |

^a^ Sequence type (ST) and clonal complex (CC), ^b^ CDS stands for coding sequences, ^c^ recently identified in the new version of the genome (Okumura *et al*. 2018)

**References:**

Okumura, K., Arai, R., Okura, M., Kirikae, T., Takamatsu, D., Osaki, M., et al. (2011). Complete genome sequence of *Melissococcus plutonius* ATCC 35311. *J Bacteriol* 193, 4029–30. doi:10.1128/JB.05151-11.

Okumura, K., Arai, R., Okura, M., Kirikae, T., Takamatsu, D., Osaki, M., et al. (2012). Complete genome sequence of *Melissococcus plutonius* DAT561, a strain that shows an unusual growth profile and is representative of an endemic cluster in Japan. *J Bacteriol* 194, 3014. doi:10.1128/JB.00437-12.

Okumura, K., Takamatsu. D., Okura, M. (2018) Complete genome sequence of *Melissococcus plutonius* DAT561, a strain that shows an unusual growth profile, obtained by PacBio sequencing. *Genome Announc* 6: e00431-18. doi:10.1128/genomeA.00431-18.

**Table S3.** **CheckM results for *M. plutonius* genome completeness, based on the marker set for *Enterococcaceae* (with 55 genomes).**

| **Strain** | **Markers** | **Marker sets** | **0** | **1** | **2** | **3** | **4** | **5+** | **Completeness** | **Contamination** | **Heterogeneity** | **GC** | **Coding density** |
| --- | --- | --- | --- | --- | --- | --- | --- | --- | --- | --- | --- | --- | --- |
| ***M. plutonius* 119** | 542 | 226 | 16 | 526 | 0 | 0 | 0 | 0 | 94.17 | 0.00 | 0.00 | 31.09 | 80.00 |
| ***M. plutonius* 21.1** | 542 | 226 | 17 | 525 | 0 | 0 | 0 | 0 | 93.73 | 0.00 | 0.00 | 31.07 | 79.71 |
| ***M. plutonius* 49.3** | 542 | 226 | 17 | 525 | 0 | 0 | 0 | 0 | 93.73 | 0.00 | 0.00 | 31.08 | 79.72 |
| ***M. plutonius* 60** | 542 | 226 | 17 | 525 | 0 | 0 | 0 | 0 | 93.73 | 0.00 | 0.00 | 31.05 | 79.75 |
| ***M. plutonius* 764-5B** | 542 | 226 | 17 | 525 | 0 | 0 | 0 | 0 | 93.73 | 0.00 | 0.00 | 31.09 | 79.89 |
| ***M. plutonius* 765-6B** | 542 | 226 | 17 | 525 | 0 | 0 | 0 | 0 | 93.73 | 0.00 | 0.00 | 31.09 | 79.86 |
| ***M. plutonius* 82** | 542 | 226 | 16 | 526 | 0 | 0 | 0 | 0 | 94.17 | 0.00 | 0.00 | 31.06 | 79.87 |
| ***M. plutonius* 90.0** | 542 | 226 | 16 | 526 | 0 | 0 | 0 | 0 | 94.17 | 0.00 | 0.00 | 31.12 | 80.02 |
| ***M. plutonius* ATCC 35311** | 542 | 226 | 18 | 524 | 0 | 0 | 0 | 0 | 93.92 | 0.00 | 0.00 | 31.24 | 79.16 |
| ***M. plutonius* B5** | 542 | 226 | 17 | 525 | 0 | 0 | 0 | 0 | 93.73 | 0.00 | 0.00 | 31.11 | 80.02 |
| ***M. plutonius* DAT561** | 542 | 226 | 16 | 526 | 0 | 0 | 0 | 0 | 94.26 | 0.00 | 0.00 | 31.22 | 79.38 |
| ***M. plutonius* H6** | 542 | 226 | 17 | 525 | 0 | 0 | 0 | 0 | 93.73 | 0.00 | 0.00 | 31.07 | 79.68 |
| ***M. plutonius* L9** | 542 | 226 | 17 | 525 | 0 | 0 | 0 | 0 | 93.73 | 0.00 | 0.00 | 31.07 | 79.83 |
| ***M. plutonius* S1** | 542 | 226 | 17 | 525 | 0 | 0 | 0 | 0 | 93.73 | 0.00 | 0.00 | 31.23 | 79.16 |
